# How Micronutrient Status May Affect Eating Behavior—Hypothesis and Perspectives

**DOI:** 10.3390/nu18040594

**Published:** 2026-02-11

**Authors:** Wahebah Alanazi, Caroline Allen, Nori Geary, Ailsa Marsh, Jeffrey M. Brunstrom, Peter J. Rogers, Richard D. Mattes, Hans-Rudolf Berthoud, Fred Provenza, Gareth Leng, Mark Schatzker, Sarah Lewis, Adrian Holliday, Kirsten Brandt

**Affiliations:** 1 Department of Food and Nutrition Sciences, Faculty of Science, University of Tabuk, Tabuk 71491, Saudi Arabia; 2 Department of Psychology, Durham University, Durham DH1 3LE, UK; 3 School of Psychology, Newcastle University, Newcastle upon Tyne NE2 4DR, UK; caroline.allen@newcastle.ac.uk; 4 Department of Psychiatry, Weill Cornell Medical College, New York, NY 10065, USA; 5 Department of Social Work, Education & Community Wellbeing, Northumbria University, Newcastle upon Tyne NE1 2SU, UK; ailsa.s.marsh@northumbria.ac.uk; 6 NIHR Bristol Biomedical Research Centre, University Hospitals Bristol, Bristol BS8 2BN, UK; jeff.brunstrom@bristol.ac.uk; 7 Weston NHS Foundation Trust, University of Bristol, Bristol BS1 3NU, UK; 8 Nutrition and Behaviour Unit, School of Psychological Science, University of Bristol, Bristol BS8 1TU, UK; peter.rogers@bristol.ac.uk; 9 Department of Nutrition Science, College of Health and Human Sciences, Purdue University, West Lafayette, IN 47907, USA; mattes@purdue.edu; 10 Neurobiology of Nutrition and Metabolism Department, Pennington Biomedical Research Center, Louisiana State University System, Baton Rouge, LA 70808, USA; berthohr@pbrc.edu; 11 Department of Wildland Resources, Utah State University, Logan, UT 84322, USA; fred.provenza@emeriti.usu.edu; 12 Centre for Discovery Brain Sciences, University of Edinburgh, Edinburgh EH16 4SB, UK; gareth.leng@ed.ac.uk; 13 Modern Diet and Physiology Research Center, McGill University, Montreal, QC H4A 3J1, Canada; 14 Bristol Medical School, Bristol Population Health Science Institute, University of Bristol, Bristol BS8 2PS, UK; s.j.lewis@bristol.ac.uk; 15 MRC Integrative Epidemiology Unit, University of Bristol, Bristol BS8 2BN, UK; 16 School of Biomedical, Nutritional, and Sport Science, Faculty of Medical Sciences, Newcastle University, Newcastle upon Tyne NE2 4HH, UK; adrian.holliday@newcastle.ac.uk; 17 Human Nutrition & Exercise Research Centre, Population Health Sciences Institute, Newcastle University, Newcastle upon Tyne NE2 4HH, UK

**Keywords:** essential vitamins and minerals, food choice, homeostasis, negative-feedback control, learned food preference, nutrient status, intuitive eating

## Abstract

The importance of micronutrient status in human food choice remains a fundamental issue needing further investigation. The objectives of the present paper are to present and discuss historic and current research together with a general model incorporating this interaction and to suggest future research to address the questions this poses. By definition, essential nutrients must be consumed in sufficient amounts to meet an individual’s requirements. While data indicate that complex neuroendocrine mechanisms provide negative-feedback control of energy and protein intake to support homeostasis, corresponding mechanisms controlling micronutrient intake are less well studied. In some contexts, they are explicitly assumed to be absent, specifically for models evaluating safety and risks of deficiencies. However, it may be hypothesized that for at least some micronutrients, mechanisms exist that aid attainment of requirements by altering preference for micronutrient-rich foods so as to increase ingestion of foods containing them, similar to how being thirsty increases the appeal of watermelon compared with dry food. If this hypothesis is correct, it may hold important implications for understanding the types and quantities of foods ingested. Greater appeal in foods richer in essential nutrients may reduce the risk of malnutrition. However, by extension, it may be posited that the use of supplements could confound the most healthful food choices. For example, obtaining vitamin C from supplements or fortified foods could then causally reduce the dietary intake of vegetables and fruits by reducing the appeal of these foods. The unintended consequence may be a lower intake of fiber, nitrate, and phytochemicals, food constituents that may contribute to health without being essential nutrients themselves. This hypothesis can and should be tested empirically, for example, through randomized placebo-controlled supplementation trials. If clear causal effects are documented, clinical and public health guidance will require critical evaluation and possible modification.

## 1. Introduction

### 1.1. The Concept of Homeostatic Control of Food Intake

The term homeostasis was coined by Cannon in 1929 as a refinement of Bernard’s 1878 notion of the constancy of the internal environment, as reviewed by Geary [1]. Homeostatic control of nutrient intake that purportedly drives food choices to ensure nutrient needs are met is implied as an aspect of several partially overlapping concepts that can be found in the literature: ‘Self-selection’ and ‘Nutrient-specific hungers’ [2], ‘Nutritional wisdom’ [3], ‘Intuitive eating’ [4], ‘Energy balancing’ [5], ‘Nutrient balancing’ [6,7], ‘Nutritional intelligence’ [8], and weight and adiposity regulation [9]. Whilst these concepts differ as to which variables are homeostatically regulated and how homeostasis is achieved, they have in common the notion of negative-feedback control, with a more or less flexible reference level for the regulated variable. They also all recognize that cooperation between behavior and metabolic regulation is fundamental to homeostasis of nutrient status. In a different context, the ability of early humans to recognize (develop a preference for) novel sources of nutritious food and incorporate them into their food culture (cuisine) may partly explain their survival during periods of pre-history where all other hominin species became extinct [10].

### 1.2. Models and Mechanisms of Homeostatic Influences on Energy and Macronutrient Intake

Regarding intake of energy and of protein, a degree of negative-feedback control (intake being affected by current status vis a vis requirement) is generally accepted [11,12,13]. For example, on a timescale of months or longer, increased energy requirements, caused, for example, by pregnancy or very high physical activity, are accompanied by corresponding increased appetite and food intake, which typically reverses when the requirement is reduced. Extensive research suggests that appetite is modulated by a complex network of physiological, psychological, social, and environmental factors that influence the motivation to initiate an eating event (meal frequency), terminate an eating event (meal size), and food choice, as detailed in recent reviews [14,15]. However, most research in this area focuses on understanding the energy imbalance that can lead to obesity [16], and in that context, a person’s appetite on a given occasion cannot be predicted from a simple homeostatic model, as explained in [5,17].

### 1.3. Is Micronutrient Intake Affected by Micronutrient Requirements?

**Figure 1:**
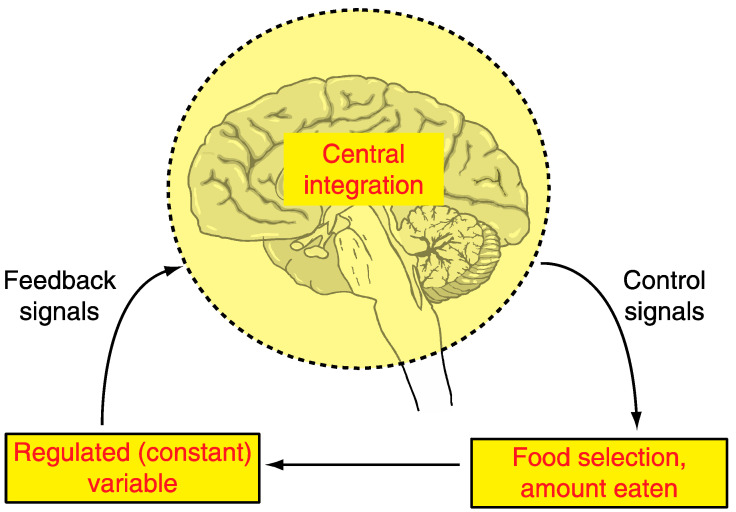
Schematic diagram of the organization of a negative-feedback control system, such as is thought to regulate micronutrient homeostasis and energy homeostasis. An afferent feedback signal related to the regulated variable is compared by the brain to the desired level of the signal and to other inputs. This integrated signal then causes the brain to initiate or adjust effector signals that affect control of the selection of food, amount eaten, energy expenditure, etc., in ways that bring the regulated variable back to the appropriate level, that is, to reestablish homeostasis (from [20], with permission).

The 2000 Institute of Medicine report on Dietary Reference Intakes [11] stated that in contrast to energy, intakes of non-energy yielding nutrients (micronutrients) are independent of the corresponding requirements (no homeostatic control). Despite substantial refinements in the understanding of appetite control since then (reviewed by Carreiro et al. [18] and Watts et al. [15]), it appears that no subsequent publications have directly and quantitatively addressed this apparent distinction between macro- and micronutrients. Only two reviews (Berthoud et al. [19] and Geary [20]) have presented the homeostatic control of energy and micronutrients in humans as conceptually identical (Figure 1).

Detailed animal studies have demonstrated homeostatic control of sodium status, including control of water and salt intake [21], and similar negative-feedback control of intake has been reported for protein [22], calcium [23], and vitamin C [24]. Data from observational studies are consistent with the idea that negative feedback control also affects human food choices in contexts where this is not simply implementation of dietary advice. For example, Brunstrom and Schatzker [25] used food pictures to show that combinations of food choices that improve overall nutritional balance were preferred; increased intake of salt in Addisons’s disease [2] was associated with increased sensitivity to the taste of salt [26]. However, it does not appear that any quantitative experimental investigations explicitly addressing feedback-regulated control of micronutrient intake (other than sodium) in humans have been published since before World War II.

**Scheme 1:**
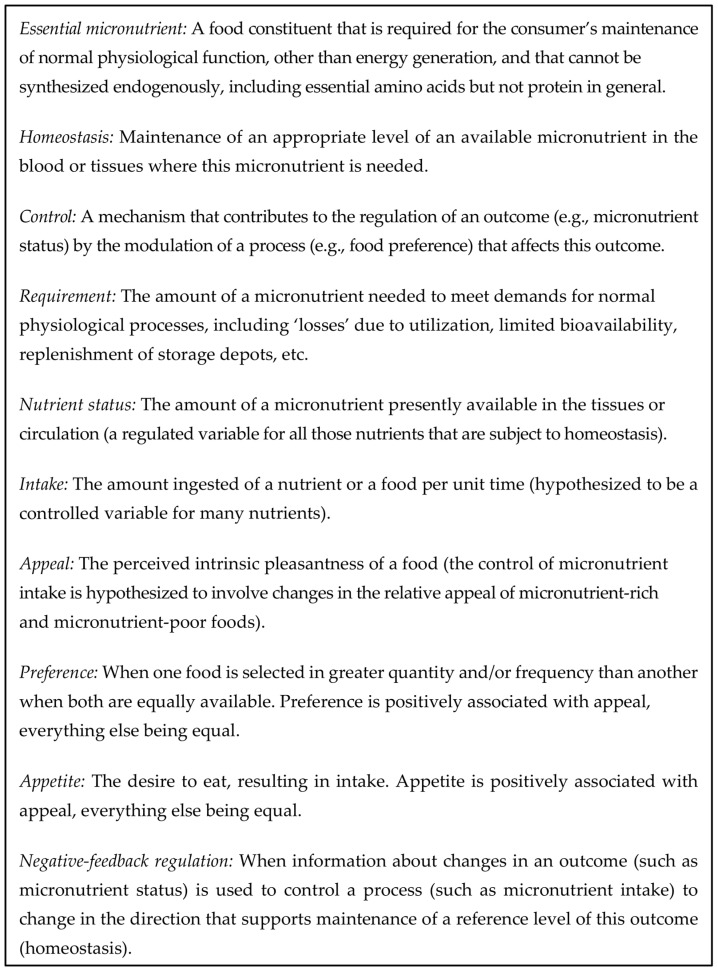
General conceptual definitions, developed by discussion among the authors, for the present context (micronutrient homeostasis).

The objectives of this article are to present a hypothetical model for how negative-feedback regulation of micronutrient status may have important effects on human food choice, at least in certain situations, with potentially wide-ranging consequences, to briefly review historic and recent relevant research, and to outline some potential tests of this hypothesis (Scheme 1).

## 2. Previous Concepts and Research, Historic Context

Soon after vitamins and minerals were discovered to be essential nutrients early in the 20th century, researchers started to investigate whether animals [2,27] and human infants [28] are able to select diets that provide sufficient intake of all the studied micronutrients and how such selection might operate. Different approaches focused on either physiologically determined innately recognized ‘specific hungers’ [2], as for sugar or sodium, or how learned associations between food flavors and micronutrient content might shape adequate nutritional choices [27]. However, the hypotheses tested in rat models often contrasted with practical observations and concerns in animal husbandry, such as farm animals consuming unhealthy amounts of concentrate feeds if given the opportunity [29]. Subsequently, priorities for experimentation with humans changed, along with improved ethical standards [30], and ‘variety seeking’ (reduced preference for any recently consumed food) started to be considered the primary mechanism to ensure ingestion of a good balance of nutrients [31]. The observed inconsistencies between different experimental designs and concepts were interpreted as teleological biases undermining the entire concept of homeostatic nutrient intake control [32], and the paucity of data linking individual micronutrient requirements to intake in humans was interpreted as an absence of effect [11].

The research on macronutrient intake control has passed through stages with increasingly complex models. Initially (until around 2000), the main emphasis was on physiological aspects of feedback regulation. As the obesity epidemic gained pace, research on factors affecting voluntary food intake increasingly focused on understanding how some people become obese or under-nourished [18]. Regarding micronutrients, later literature reviews on human food choice rarely mentioned earlier concepts (e.g., [33]) regarding the possibility that micronutrient status might affect food intake preferences [34,35]. This also applied to reviews focused on food choice in the evolutionary context [36], on balancing nutrient intake with nutrient requirements [7], or on human ability and preference to select foods with optimal nutrient composition [8]. Two recent reviews [37,38] explicitly state that humans have not evolved homeostatic feedback mechanisms to control micronutrient intake, even though no data or other evidence for this absence are specified in these reviews. In contrast, three books describe how a connection between micronutrient requirements and intake in animals could be relevant in human nutrition [39,40,41]. For example, one of them explained how mixing vitamins into pig rations caused the pigs to consume less vegetation [42] and suggested that human food fortification may have a similar effect on vegetable intake [41]. However, these books do not seem to have influenced current thinking in the nutrition research community. No research intended to assess the presence or absence of moderate (i.e., ecologically relevant) associations between an individual’s requirements and their intake of micronutrients is apparent in the literature (although it must be acknowledged that the lack of methods for determining nutrient requirements of individuals precludes testing this directly). It appears as if an absence of data caused a lack of awareness, resulting in relevant data not being collected, further eroding awareness and so on.

Interestingly, in the earliest studies on diet choice in animals, some factors causing apparent inconsistencies were identified and explained. By 1933, Harris and colleagues had identified ways to ‘deceive’ rats into unhealthy choices [27], e.g., by presenting them with too many choices or by unpredictably changing the flavor–nutrient associations among the food options, resulting in vitamin deficiency even though food containing sufficient amounts of the vitamin was available. Such observations do not necessarily (as previously claimed by Galef [32]) invalidate the hypothesis that micronutrient intake is affected by requirements. On the contrary, by demonstrating some limits to regulation, they may be particularly significant in today’s varied and inconsistent ‘foodscape’ [40], including the conundrum of how multiple-ingredient (ultra-processed) foods may affect human health [43].

## 3. Proposed Concept and Model

### 3.1. Core Elements of Concept

**Figure 2:**
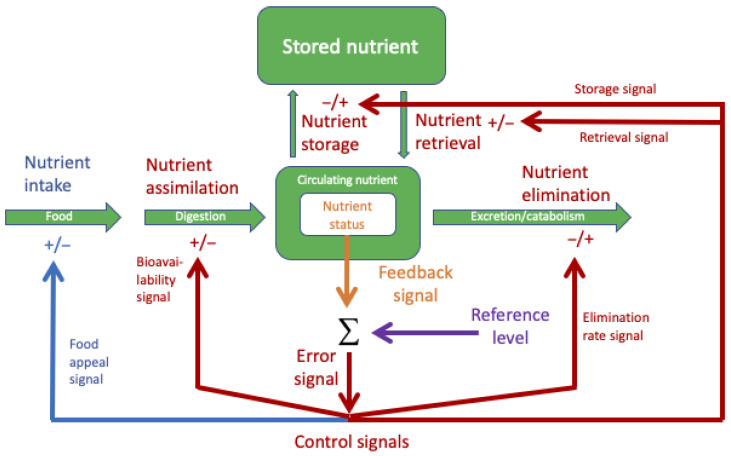
Systems diagram to illustrate the concept of negative-feedback regulation of micronutrient status. Shown in green is the movement of nutrient molecules, from entering the body in food to their presence in the circulation or tissues, their sequestering in storage, and their elimination from the body via excretion or catabolism. Micronutrient status depends on the rates of intake, assimilation, storage/retrieval, and elimination. Micronutrient status is sensed (orange) and this feedback signal compared (Σ) with the reference level (purple); the difference (error signal, red) generates the control signals, which affect one or more of these rates, depending on the nutrient. Importantly, the control signals are differentially generated (i.e., each has its own feedback–gain process) so may have different thresholds, different intensities, etc. For most micronutrients, there is already evidence for negative-feedback control of some or all of the **internal processes** (red arrows); this maintains a relatively constant nutrient status (homeostasis) despite excess or short-term fluctuations in the nutrient’s intake and minimizes the consequences of deficiency. However, if negative feedback from nutrient status also affects **behavioral processes**, i.e., nutrient intake, as has been shown in various animals, then it is appropriate to suggest (and investigate) the idea that control of human nutrient **intake** (blue arrow) also contributes to micronutrient homeostasis.

To summarize the discussion above, an enhanced preference for nutrient-rich foods in response to an insufficient supply of the corresponding nutrient in the overall diet is a key prerequisite for feedback control to increase intake (see Figure 2). While the present paper focuses on micronutrients, the concept described below is not specific to any given nutrient. Indeed, it is compatible with several published (macro)nutrient intake control models, such as energy balancing [5], nutritional geometry [6], body weight and adiposity regulation [9], sodium homeostasis [21], and the framework of feeding behavior [44].

The figure depicts both increases and decreases in the ‘Food appeal signal’. In the former case, low nutrient status leads to increased preference for and intake of nutrient-containing foods, as has been found for several micro- and macronutrients in animal studies [22,24]. In the latter case, excessively high nutrient status leads to decreased preference for foods containing this nutrient, as has been observed for sodium and protein [21,45] and indicated by excessively high micronutrient content in meals occurring significantly less frequently than random chance [25].

**Figure 3:**
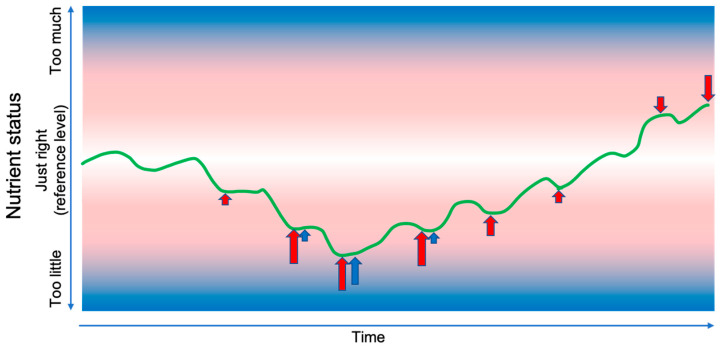
Illustration of anticipated effects of feedback control of internal processes and intake on nutrient status homeostasis. If the intake of a nutrient during a period of time is so low that the control of internal processes (red) becomes insufficient to maintain the nutrient status (green) near the reference level (white background), then the nutrient status reaches such a low level that intake control (blue) may take effect (see Figure 2). Arrows indicate the type and intensity of control signals elicited at various levels of nutrient status, their direction, length and colors reflect the intensity of each type of control signal, and the background color saturation indicates the deviation from the reference level. Depicting the control signals as a series of discrete events is deliberately simplified; the effects are more likely to be continuous, with variable intensity.

Several internal homeostatic mechanisms, including changes in bioavailability, storage, and excretion) fine-tune the effective nutrient status (i.e., concentrations in tissues or blood). Due to these internal controls, behavioral feedback control of intake may operate only intermittently, requiring a larger threshold of deviation from the reference level than the internal mechanisms (see Figure 3), and behavioral controls may involve substantial lag and hysteresis. Finally, it is likely that not all the negative-feedback controls shown exist for every micronutrient.

### 3.2. Possible Mechanisms for Intake Control

For such a scheme to operate, a mechanism must exist to distinguish between rich and poor sources of the regulated nutrient. While some nutrients, such as sugar, sodium, glutamate [46], and possibly calcium [47], correspond with specific taste receptors, the sense of taste is far from sufficiently accurate to directly sense even macronutrient composition directly [48], and most micronutrients have no known specific taste receptor. However, animal experiments have clearly demonstrated a second option, ‘flavor–nutrient learning’, for micronutrients, to develop a heightened appeal of a characteristic flavor of a food that contains a needed micronutrient [49]. This is an ability that humans may share. For example, experimentally imposed low or high protein intake affected subsequent protein intake and preference for savory flavors in women [45], even though the results of efforts to assess flavor–nutrient learning for energy-yielding macronutrients in humans have been inconsistent in other human studies [50,51]. A third potential route could be through modulation of non-nutrient-specific ‘variety seeking’ [31], where a low nutrient status could enhance the propensity for consumption of unfamiliar foods in general and thus increase the opportunity to alleviate an insufficient supply of a nutrient in the habitual diet. Overall food deprivation in mice affects specific neurons that increase the motivation to seek food, even in the presence of a predator [52]. While this study addressed total food intake rather than a specific nutrient, Yuan et al. [53] reported that deficiency in any one of eight essential amino acids activated the same neuronal pathway to increase explorative behavior in stressed mice, although these authors interpreted this as an ‘antidepressant effect’ of leucine deprivation (illustrating the lack of awareness suggested above).

These three mechanisms (taste sensing, flavor–nutrient learning, and modulation of variety seeking) are not mutually exclusive but may all operate together in the control of the same nutrient.

However, paraphrasing a paper by Forbes and Kyriazakis [54], if food appeal in humans is affected by negative-feedback regulation, why do we not always choose wisely? There are several potential answers to this question:

- If the content of a nutrient in the habitual diet is sufficiently high and stable to allow the status of this nutrient to stay within the internally regulated zone (red background in Figure 3), then this nutrient may not affect food preferences/intake at all—this may apply to most nutrients in most diets.
- If the content of a nutrient in the habitual diet is variable, for example, changing with seasons of the year, then food rich in this nutrient will become more preferred during nutrient-depleted periods than during periods of plenty. The magnitude of this effect, and how quickly it appears once sub-optimal levels occur, will depend on the capacity for storage, which varies with the nutrient.
- If an individual has never previously experienced borderline deficiency (the lower blue band in Figure 3) of a nutrient, then if it does occur, they will not immediately be able to adjust their intake; they first must learn which foods counteract the nutrient imbalance. If such foods are not encountered at this stage, the imbalance may persist and cause harm.
- After having experienced one or more episodes of nutrient imbalance and learned how to counteract it, presumably the response to future episodes will be faster and more targeted—as found for farm animals [54].

Note that some animal studies indicate that nutrient-related preferences may manifest as an aversion to nutrient-poor foods [55], even in the absence of a nutrient-rich preferred alternative (in which case, it is indistinguishable from loss of appetite). This may also apply to humans; it is possible that such reduced preference for a nutritionally inadequate habitual diet is experienced as increased variety seeking.

## 4. Proposed Research and Implications

### 4.1. Testing the Hypothesis—Is Micronutrient Intake Affected by Requirement?

Ethical considerations [30] limit what nutrient manipulations are acceptable in future human studies; however, some options are still possible. The response to repeated intake of a distinctive food providing micronutrients to individuals at risk of deficiency could be recorded without ethical concerns, particularly if combined with objective measures of nutritional status. However, this would not be expected to show any effect in populations where deficiency problems are rare. Studies focusing on food appeal and behavior without changing the participants’ diets (as in the study by Brunstrom & Schatzker [25]) are not ethically problematic and could involve volunteers who experience predictable and quantifiable changes in their requirements, such as pregnancy, lactation, blood donation, or surgery. Some pathologies such as celiac disease are associated with micronutrient deficiencies [56], which are resolved after treatment, so changes in selected food appeal before and after treatment could detect changes associated with improved nutritional status. Observational studies could support or contradict the hypothesis but are not sufficient to demonstrate causality, due to the risk of unrecognized confounding factors. Potentially, for certain micronutrients, Mendelian randomization designs could be used. These use genetic variants related to micronutrient sufficiency (i.e., those involved in the metabolism of specific micronutrients) as exposure variables and investigate the putatively causal effect of this on micronutrient intake as the outcome. This has conceptual similarity with the previously mentioned animal study [24], where a rat strain with a genetically determined higher than normal vitamin C requirement was used to demonstrate negative-feedback control of intake. Intriguingly, a 2006 study with data on MTHFR (C677T) genotypes and on intake of methionine and B-vitamins in 1492 people [57] found a 10% higher intake of vitamin B2 (a co-factor for the MTHFR enzyme) in 124 people with the TT genotype (with lower enzyme activity) compared with those with CC or CT genotypes. While this small sample did not yield sufficient statistical power to detect significance, the larger datasets available today could be analyzed to find out if this trend is reproducible. Another approach could be to identify genetic loci associated with intake of specific micronutrients and therefore likely to have a role in their regulation, as has recently been achieved for macronutrients [58].

Another experimental option is to reduce the requirement, as in a randomized placebo-controlled trial (RPCT) of micronutrient supplementation. Some relevant data may already exist, from published studies that (for any reason) combined a micronutrient RPCT with assessment of habitual dietary intake, and could be analyzed for effects of supplementation on intake [59]. For example, our hypothesis predicts that supplementation with vitamin C will reduce the intake of vitamin C-rich foods such as vegetables and fruits by preventing this micronutrient from being the most limiting nutrient [54]. As explained above and discussed in other reviews [8,44], the effects should not be expected to be large or rapid, particularly in populations with high food security. Therefore, researchers should initially be encouraged to carry out pilot studies with a variety of designs to estimate the sample sizes required to test this hypothesis.

### 4.2. Consequences if This Concept Has Significant Effect on Food Choices

#### 4.2.1. Regarding Populations at Risk of Micronutrient Deficiencies

As detailed on pages 212–224 in the Institute of Medicine’s [11] consensus report, if micronutrient intakes are in fact correlated with their requirements (individual nutritional status), then the established methods for estimating nutrient deficiency at the population level will give incorrect (too high) estimates. In other words, micronutrient malnutrition may be less widespread than expected. In this case, micronutrient provision to vulnerable populations could be made more efficient (target the individuals in greatest need) by being distributed in a food (e.g., a gum or a cookie) with a distinctive taste, rather than as tablets or injections. Individuals at risk of deficiency should then theoretically develop a preference for the taste and come back for more, while those at risk of excessive intake (page 123 in [11]) may discover that they do not like it.

#### 4.2.2. Regarding Addition of Micronutrients to Food in General

If human homeostatic intake control works in the same ways as in laboratory animals, then it may also be ‘confused/deceived’ by the same factors, specifically unpredictable contents of micronutrients and/or excessive variety of flavors [27]. If this is the case, then the control of micronutrient intake may be less accurate for humans in a modern society with thousands of foods to choose from, not to mention the existence of fortified foods and nutritional supplements, compared with a population consuming a traditional diet composed of far fewer, easily identifiable, and nutritionally distinct ingredients. There are no indications that modern diets (as defined above) cause more micronutrient deficiencies than traditional diets. However, even a small change in food choice preferences could have substantial health consequences in the long run, if it systematically reduces the relative intake of foods with high micronutrient density, such as vegetables, fish and whole-grain cereals, since these are exactly the foods that are associated with good health [60,61]. Micronutrient-dense foods are also major dietary sources of non-essential dietary constituents such as fiber, phytochemicals [62], and nitrate [63], which were present in excess of optimal intake in ancient and pre-human diets and therefore presumably were not subject to natural selection for feedback control of low intake. While people who consume micronutrient supplements are obviously not at risk of deficiency in the supplemented nutrients, feedback regulation could lead to reduced intake of such other important dietary constituents, which may be at least as important for the differential effect of a healthy diet as the essential micronutrients themselves [64,65].

If future research confirms such a relation, then this should be taken into account when evaluating the risk/benefit balance of non-targeted fortification or supplementation with micronutrients.

### 4.3. Conclusion and Recommendations

Homeostatic negative-feedback control of micronutrient intake may affect human dietary habits. The concept can be represented in a model, which can guide the formulation of testable hypotheses. If this effect is substantial, it would be sufficiently important for public health that it is worthwhile to investigate. Researchers are recommended to consider negative-feedback regulation of micronutrient status as a possibility to take into account whenever planning or analyzing research that involves changes in micronutrient intake (whether depletion or supplementation) in humans or animals. Specifically, food intake information should routinely be collected both during and after such interventions, to allow for testing for corresponding effects on the habitual diet.

## Data Availability

No new data were created or analyzed in this study.

## Abbreviation

The following abbreviation is used in this manuscript:

RPCT	Randomized placebo-controlled trial
